# Clinical efficacy of T-cell therapy after short-term BRAF-inhibitor priming in patients with checkpoint inhibitor-resistant metastatic melanoma

**DOI:** 10.1136/jitc-2021-002703

**Published:** 2021-07-01

**Authors:** Troels Holz Borch, Katja Harbst, Aynal Haque Rana, Rikke Andersen, Evelina Martinenaite, Per Kongsted, Magnus Pedersen, Morten Nielsen, Julie Westerlin Kjeldsen, Anders Handrup Kverneland, Martin Lauss, Lisbet Rosenkrantz Hölmich, Helle Hendel, Özcan Met, Göran Jönsson, Marco Donia, Inge Marie Svane

**Affiliations:** 1 National Center for Cancer Immune Therapy, Department of Oncology, Herlev University Hospital, Herlev, Denmark; 2 Department of Oncology, Clinical Sciences, Lund University, Lund, Sweden; 3 Department of Plastic Surgery, Herlev University Hospital, Herlev, Denmark; 4 Department of Clinical Medicine, University of Copenhagen, Copenhagen, Denmark; 5 Department of Clinical Physiology and Nuclear Medicine, Herlev University Hospital, Herlev, Denmark; 6 Department of Immunology and Microbiology, University of Copenhagen, Copenhagen, Denmark

**Keywords:** immunotherapy, adoptive, lymphocytes, tumor-infiltrating, melanoma, clinical trials as topic

## Abstract

**Purpose:**

Despite impressive response rates following adoptive transfer of autologous tumor-infiltrating lymphocytes (TILs) in patients with metastatic melanoma, improvement is needed to increase the efficacy and broaden the applicability of this treatment. We evaluated the use of vemurafenib, a small-molecule BRAF inhibitor with immunomodulatory properties, as priming before TIL harvest and adoptive T cell therapy in a phase I/II clinical trial.

**Methods:**

12 patients were treated with vemurafenib for 7 days before tumor excision and during the following weeks until TIL infusion. TILs were grown from tumor fragments, expanded in vitro and reinfused to the patient preceded by a lymphodepleting chemotherapy regimen and followed by interleukin-2 infusion. Extensive immune monitoring, tumor profiling and T cell receptor sequencing were performed.

**Results:**

No unexpected toxicity was observed, and treatment was well tolerated. Of 12 patients, 1 achieved a complete response, 8 achieved partial response and 3 achieved stable disease. A PR and the CR are ongoing for 23 and 43 months, respectively. In vitro anti-tumor reactivity was found in TILs from 10 patients, including all patients achieving objective response. Serum and tumor biomarker analyses indicate that baseline cytokine levels and the number of T cell clones may predict response to TIL therapy. Further, TCR sequencing suggested skewing of TCR repertoire during in vitro expansion, promoting certain low frequency clonotypes.

**Conclusions:**

Priming with vemurafenib before infusion of TILs was safe and feasible, and induced objective clinical responses in this cohort of patients with checkpoint inhibitor-resistant metastatic melanoma. In this trial, vemurafenib treatment seemed to decrease attrition and could be considered to bridge the waiting time while TILs are prepared.

## Introduction

The introduction of checkpoint inhibitors for metastatic melanoma (MM) has vastly improved patient outcomes^1–4^ and provided long-term benefit in a subset of patients.^5^ Despite this, the majority of patients progress due to primary or acquired immune resistance and succumb to their cancerous disease. Thus, research on new treatments or combinations hereof is still highly warranted.

In MM, adoptive cell transfer (ACT) using tumor-infiltrating lymphocytes (TILs), following a preconditioning non-myeloablative chemotherapy regimen and subsequent interleukin-2 (IL-2) treatment, has consistently induced objective clinical responses in 40%–50% of treated patients in multiple phase I/II trials, with complete response (CR) rates of up to 20%.^6–9^ The majority of patients achieving a CR experiences long-term benefit^9 10^; perhaps even cures. However, manufacturing TILs for infusion takes on average 4–6 weeks using the young TIL method.^11 12^ In those weeks, the patient receives no anti-cancer treatment and a substantial fraction of patients deteriorate quickly and, thus, will be prevented from receiving a potentially life-saving treatment.^6 7^

Small molecule BRAF inhibitors (BRAFi) were introduced in the early 2010s to treat the 40%–50% of patients with MM bearing BRAF-mutated tumors. It became evident that although treatment with BRAFi resulted in high objective response rates (ORRs) in the range of 50%–60% with rapid tumor regression, the effect was transient, with a short median progression-free survival (mPFS) of 7 months,^13 14^ because resistance to BRAFi arises quickly.^15^ With the addition of a MEK inhibitor, the response rates and overall survival (OS) were further improved, but durable responses are rare and mPFS is only 11 months,^16 17^ leaving a large space of unmet need.

In addition to the direct anti-cancer effects, vemurafenib (vem), a BRAFi, has immunomodulatory effects; increased CD8^+^ T cell infiltration during treatment with vem,^18 19^ downregulation of VEGF,^20^ a less immune-suppressive tumor microenvironment due to abrogated secretion of interleukine-1α/β, −6 and −10 and downregulation of programmed death-ligand 1 and −2 (PD-L1 and −2) by tumor-associated fibroblasts.^21^ Furthermore, treatment with vem increases tumor antigen expression and anti-tumor reactivity of CD8^+^ T cells, both in vitro and in vivo.^20 22^ Indeed, the addition of vem to ACT has shown synergistic effects in mouse models,^20 23 24^ enforcing the rationale for clinical testing.

The ability to combine the fast and frequent response to BRAFi with the long-term efficacy of immunotherapy might dramatically change the natural history of BRAF-mutant melanoma. In this study, we set out to address this issue by initially investigating the combination of ACT using TILs with vem.

## Materials and methods

### Clinical trial design

The clinical trial was conducted as a phase I/II non-randomized trial and was planned to include up to 12 patients. Patients were treated at the National Center for Cancer Immune Therapy and Department of Oncology, Herlev University Hospital (Herlev, Denmark, ClinicalTrials.gov identifier: NCT02354690).

The primary objective was to evaluate the safety and feasibility of the study treatment. Secondary objectives were to determine ORR by response evaluation criteria in solid tumors (RECIST) V.1.1,^25^ progression-free survival (PFS), OS and immunological responses.

### Patients

Eligible patients were between 18–70 years of age, had histologically confirmed diagnosis of MM harboring a mutation in the BRAF gene, American Joint Committee of Cancer stage III or IV, with tumor available for surgical removal *and* at least one additional measurable lesion according to RECIST V.1.1, had an Eastern Cooperative Oncology Group performance status of 0–1,^26^ had a life expectancy >3 months, and adequate organ function. Main exclusion criteria were uveal melanoma, untreated or symptomatic brain metastasis, a history of autoimmune disease or chronic infections, and prior treatment with BRAFi.

### Treatment overview

After screening and inclusion, patients started vem 960 mg orally two times a day 7 days before surgery and until hospitalization. During hospitalization patients received cyclophosphamide 60 mg/kg/day (day −7 and −6) and fludarabine 25 mg/m2/day (day −5 to −1) as previously described.^6 27^ TILs were infused on day zero followed by continuous intravenous infusion of IL-2 6–8 hours later. IL-2 was administered according to the decrescendo regimen; 18 MIU/m^2^ over 6 hours, 18 MIU/m^2^ over 12 hours, 18 MIU/m^2^ over 24 hours, and 4.5 MIU/m^2^ over 24 hours repeated three times. Maximum dose of IL-2 administered was 135 MIU as 2 m^2^ was set as the maximum body surface area. The treatment and monitoring schedule is shown in online supplemental figure S1.

10.1136/jitc-2021-002703.supp1 Supplementary data

Prophylactic antibiotics, antiemetics, granulocyte colony stimulating factor and other supportive treatment were administered as previously described.^6^ Dose reductions of vem and IL-2 followed standard guidelines.

### Toxicity

Toxicity was assessed using Common Terminology Criteria for Adverse Events version 4.0. Assessments were performed continuously, and as minimum at baseline, 4 weeks after vem treatment initiation, daily during hospitalization and at subsequent follow-up visits.

### Clinical efficacy and patient samples

Clinical efficacy was assessed by Flour-18-deoxyglucose-positron emission tomography/CT (FDG-PET) scans at baseline, before hospitalization, at week 6 and 12 after TIL infusion and every third month thereafter until disease progression. Objective responses were evaluated according to RECIST V.1.1.

Blood samples for immune monitoring were collected at baseline, before hospitalization, at discharge and at following evaluation visits. Blood was collected in heparinized tubes and was kept for a maximum of 4 hours until handling according to standard operating procedure. In brief, peripheral blood mononuclear cells (PBMCs) were separated using centrifugation on a Lymphoprep (Takeda, Roskilde, Denmark) density gradient. Vials of PBMCs were cryopreserved in medium containing 90% heat inactivated human AB serum (HS; Sigma Aldrich) and 10% dimethyl sulfoxide using controlled-rate freezing (Cool-Cell, Biocision) in a −80°C freezer and the next day moved to a −140°C freezer until further processing.

Serum samples were collected before TIL infusion, 2 hours after TIL infusion and every second day until discharge. Within maximum 4 hours, serum tubes were spun at 3000*g* for 10 min. Serum was aliquoted and immediately transferred to a −80°C freezer and subsequently stored at −140°C until further processing.

### Generation of TILs

Surgically removed tumors were minced mechanically into 1–3 mm^3^ fragments and placed in 24-well culture plates (Nunc, Roskilde, Denmark) containing 2 mL culture medium consisting of 90% RPMI 1640 (Thermo Fisher Scientific), 10% HS, penicillin/streptomycin and fungizone (Bristol-Meyers Squibb) and 6000 IU/mL IL-2 (Proleukin, Novartis, Bazel, Switzerland). The plates were incubated in 37°C humidified atmosphere with 5% CO_2_. Half the medium was replaced at day 5 and subsequently three times weekly. TILs were propagated following the minimally cultured or “young TIL” method as previously described.^12^ Initial expansion was successful when pooled cultures reached >50×10^6^ total cells. The pooled TILs were either cryopreserved or directly propagated following the rapid expansion protocol (REP). In the REP 20×10^6^ cells were cultured in 80% RPMI-1640 with 10% human serum/20% AIM-V medium containing 6000 IU IL-2 and irradiated (40 Gy) allogeneic feeder cells (PBMCs from at least three different buffy coats) in a ratio of 1:200. The REP was initiated in static culture flasks and transferred to the dynamic Wave bioreactor system (GE healthcare) as previously described.^28^ At day 14 of the REP, TILs were harvested, transferred to an infusion bag and immediately administered to the patient. Sterility testing and microbiological control were performed on all TIL cultures prior to REP and infusion.

### Generation of autologous tumor digests and tumor cell lines

Autologous tumor digests (TDs) were established from the same tumor lesion from which the TILs were generated. TD was obtained from fresh tumor fragments after overnight incubation with enzyme cocktails containing 1 mg/mL collagenase type IV (Sigma Aldrich) and 0.0125 mg/mL dornase alpha (Pulmozyme, Roche). The obtained single-cell suspension was passed through 70 µm strainers and cryopreserved immediately without further analysis.

### Phenotype analyses of TILs

For phenotype analysis, cryopreserved TILs were thawed and rested overnight in RPMI 1640 containing 10% HS. TILs were harvested, washed and resuspended in phosphate-buffered saline (PBS; Lonza, Basel, Switzerland) containing 0.5% bovine serum albumin (BSA; Sigma-Aldrich). TILs were stained in the dark at 4°C for 30 min, washed and resuspended in PBS and analyzed immediately. Antibodies used were the following: CD3-HV510, CD4-PerCP, CD8-BV421 (all from BD Biosciences), and Near Infra-Red Live/dead marker (NIR) (Invitrogen).

Data acquisition was performed using a FACS Canto II flow cytometer and analyzed using FACS Diva software (both BD Biosciences).

### Anti-tumor reactivity of TILs

Assessment of anti-tumor reactivity was performed in a flow cytometry based assay staining for CD107a, intracellular interferon-γ (IFNγ) and tumor necrosis factor (TNF) as previously described.^12^ Briefly, in the presence of GolgiPlug (BD Biosciences, dilution 1:1000) and CD107a detection antibody, TILs were cocultured at 37°C for 5 hours with autologous TD at an effector to target ratio of 3:1, or alone (unstimulated sample). After coculture, cells were stained with surface antibodies and intracellular antibodies as described for phenotype analyses. In positive control wells, Staphylococcal enterotoxin B (Sigma-Aldrich) was added. The following antibodies were used: CD3-FITC, CD56-PE, CD8-PerCP, IFNγ-PE-Cy7, TNF-APC, CD107a-BV421, CD4-HV510 (all from BD Biosciences), and NIR (Invitrogen).

Tumor reactive cells were defined as cells staining double positive for any combination of CD107a, IFNγ and TNF. A positive anti-tumor response was defined as more than double the level of activity in paired unstimulated samples (background; TILs alone), at least 0.1% difference from background and a minimum of 50 positive events after subtraction of double positive events in paired unstimulated samples. In selected samples, digests alone were tested under the same coculture conditions, and we did not observe any significant spontaneous reactivity. TILs in the digests were smaller than the cultured TILs used in tumor-recognition assays and were easily gated out from the analyses; hence only cultured TILs were gated to calculate the proportion of tumor-reactive TILs.

### Serum cytokine measurement

Cytokines were measured in serum samples using a 13-plex Bio-Plex Pro Human cytokine kit (Bio-Rad Laboratories, Copenhagen, Denmark) including the cytokines IL-1β, IL-2, IL-4, IL-5, IL-6, IL-8, IL-10, IL-12(p70), IL-13, IL-17A, granulocyte-macrophage colony-stimulating factor, IFNγ and TNF. The assay was performed according to manufacturer’s instructions using a Luminex 200 and Bio-Plex manager software V.6.1 (Luminex Corporation, Austin, Texas, USA). Data analysis was performed with the STarStation V.2.3 software (Applied Cytometry Systems, Sheffield, UK).

### Nucleic acid extraction, whole exome sequencing (WES), RNA-seq and TCR-seq

DNA and RNA were extracted from baseline tumors, relapse samples (patients #09 and #13), PBMCs and TILs using AllPrep DNA/RNA Mini Kit (Qiagen). Whole exome libraries were constructed from tumor and PBMC DNA as described previously^29^ using SureSelectXT Clinical Research exome (Agilent) and sequenced on NextSeq500 to mean target depth 38x-176x (median 150x) in tumors and 69x-87x (median 75x) in MNC samples. For T-cell receptor (TCR) sequencing, TIL and tumor RNA were treated with DNAse I (Thermo Scientific) as per Illumina recommendation, and libraries were constructed from 500 ng of DNAse treated RNA using AmpliSeq Immune Repertoire Panel (Illumina) and sequenced on NextSeq500. RNA-seq was performed as previously reported.^29^ *WES data analysis*: Alignment, postalignment processing, and variant calling was performed as described^30^ using SAREK workflow.^31^ Somatic mutations were derived using Varscan as in^30^ and Strelka2 (V.2.8.2^32^;). Single nucleotide variants detected by both callers constituted the data set, while insertions and deletions from varscan were used. Mutations were annotated using Annovar^33^ and only mutations in protein coding regions and at splice sites were retained. *RNA-seq data analysis*: RNA sequencing data from bulk tumor samples were processed using Tophat2^34^ and Cufflinks^35^ as described previously.^36^ Isoform FPKM values of each gene were summed up and protein-coding genes were extracted. The data were then quantile-normalized using^37^ and log-transformed as log2(data+1). *TCR-seq data analysis*: Demultiplexed data were used to derive T cell clonotypes using MiXCR^38 39^ with the command “mixcr analyze amplicon” and the following settings: “--starting-material rna --adapters no-adapters --receptor-type TRB --region-of-interest CDR3 -−5-end no-v-primers -−3-end c-primers”. Clonotypes that were supported by fewer than 10 read counts were discarded; these constituted 10%–78% (median 54%) of all clonotypes per sample but accounted for only 0.1%–14% (median 2%) of total abundance. MiXCR output was further used to derive clonotypes metrics using VDJtools^40^ as described in.^41^ VDJtools FilterNonFunctional command was used to remove non-productive CDRs and CalcDiversityStats command was used to derive statistical metrics. For between-sample comparisons, metrics based on *resampled* data from VDJtools were used to account for different depth of sequencing between samples. Following metrics were used: “Observed Diversity” (the normalized number of clonotypes, named “Richness” throughout the manuscript for clarity and consistency with previous publications); “Normalized Shannon-Wiener index” (reflects distribution of the clonotypes within the TCR repertoire, “Evenness” throughout the manuscript); “Shannon-Wiener index” (a parameter that combines richness and evenness, “Diversity” throughout the manuscript). For comparative analyses between samples, nucleotide sequences of TCR were used. For overlap analysis of TIL and TUM clonotypes from each patient, VDJtools OverlapPair command was used to derive Morisita-Horn index. Venn diagrams were drawn using R package VennDiagram.^42^

### Statistical analysis

PFS and OS was defined as time from vem treatment initiation until progression, death of any cause, or the date of data cut-off (December 14 2018). Survival was estimated using the Kaplan-Meier method and comparisons were performed with the Mantel-Cox test. Cytokine levels at baseline were compared between groups with PFS longer or shorter than median PFS using a t-test. Comparisons of TCR data were performed using a Mann-Whitney test either paired (intrapatient comparisons) or unpaired (patient group comparisons). Correlations were described using the Pearson and Spearman tests. All statistics were computed by GraphPad Prism V.5.0 or statistical software R.

## Results

### Patient characteristics

A total of 13 patients were enrolled in the trial between November 2014 and April 2018. All patients had tumors removed and an initial TIL expansion culture was established. A brain metastasis measuring 25 mm was discovered in one patient on magnetic resonance scan before hospitalization for TIL infusion. Despite surgical and local stereotactic radiotherapy, the metastasis was not controlled, and it was deemed unsafe to treat with TIL therapy (due to high doses of IL-2). The patient was excluded from the trial and is not included in the data analyses.

Patient characteristics are summarized in table 1. Ten of twelve patients treated with TILs had stage M1c at inclusion, one had M1b and one had M1a. Patients had received a median of two prior systemic therapies, including one or more of the immune checkpoint inhibitors ipilimumab, pembrolizumab, or nivolumab administered alone or in combination with an anti-LAG3 antibody.

**Table 1:** Baseline patient characteristics

Patient	Sex/age	Primary tumor origin	Previous treatments	Time from diagnosis to ACT (y)	ECOG PS at screening	ECOG PS at admission	AJCC stage	Target lesion sum (mm)	LDH level	Metastatic sites
01	M/51	Skin	Ipi, Pem	2.3	2	0	M1c	97	Normal	SC, IM, LN, lung, liver, bone
02	M/46	Skin	Ipi, Pem	2.2	1	0	M1c	96	Elevated	SC, LN, lung, liver, adrenal glands, retroperitoneal
03	F/53	Skin	Ipi, Pem	15.2	0	0	M1c	84	Elevated	SC, IM, LN, lung
04	M/54	Skin	Ipi, Pem	3.2	1	1	M1c	44	Elevated	SC, IM, lung, liver, bone, adrenal glands
05	M/55	Skin	Ipi	3.9	1	1	M1c	136	Elevated	SC, lung, adrenal gland, bone
06	M/53	Skin	Ipi, Nivo	7.7	1	0	M1c	64	Elevated	SC, IM, lung, liver, gastric wall
07	F/50	Skin	Pem	3.7	0	1	M1b	24	Normal	LN, lung, pleura
09	F/59	Unknown	Pem, Ipi	3.1	1	0	M1c	96	Elevated	SC, lung, carcinomatosis
10	F/52	Skin	Pem	9.5	0	0	M1c	52	Elevated	LN, lung
11	M/73	Unknown	Ipi, Pem, Nivo+LAG3	0.4	0	1	M1c	147	Elevated	Skin, LN, IM, carcinomatosis, retroperitoneal
12	F/67	Skin	Pem, Ipi	3.3	0	1	M1c	67	Elevated	Skin, LN, IM, brain
13	M/54	Skin	Pem	3.9	0	0	M1a	112	Normal	SC, LN
***Median***			***2***	***3.5***	***1***	***0***				
***Range***			***(1-3)***	***(0.4–15.2)***	***(0–2)***	***(0–1)***				

ACT, adoptive cell transfer; AJCC, American Joint Committee on Cancer; ECOG PS, Eastern Cooperative Oncology Group Performance Status, F, female; IM, intramuscular; Ipi, ipilimumab; LDH; lactate dehydrogenase; LN, lymph node; M, male; Pem, pembrolizumab; SC, subcutaneous; y, years.

### Toxicity and treatment characteristics

Patients received vem for a median of 41 days (range 26–61; online supplemental table S1). Overall patient PS improved following treatment with vem. Two patients (patients 07 and 11) had worse PS due to side effects from vem treatment (most notably arthralgia and swollen joints, and nausea, respectively). Treatment-related toxicity is summarized in table 2. No unexpected toxicity was observed, and all adverse reactions were manageable following standard guidelines.

**Table 2:** Treatment related toxicity

Occurred during vemurafenib	Any grade (Number)	Grade 1–2 (Number)	Grade 3–4 (Number)
Local infection at site of surgery*	1	1	
Fever without neutropenia	2		2
Fatigue	2	2	
Myalgia/arthralgia	8	7	1
QTc prolongation	3	3	
Papilloma	1	1	
Lymphopenia	1		1
Neutropenia	1	1	
Thrombocytopenia	1	1	
Elevated liver enzymes	1	1	
Photosensitivity	5	5	
Hyperkeratosis	1	1	
Actinic keratosis	1	1	
Rash, maculopapular	7	5	2
Pancreatitis	1		1
Uveitis	1	1	
Nausea	5	5	
Oral mucositis or candidiasis	1		1
Colitis	1		1
Alopecia	1	1	
Dry skin	2	2	

Occurred during T-cell therapy or later	Any grade (Number)	Grade 1–2 (Number)	Grade 3–4 (Number)
Febrile neutropenia	12		12
Infection, verified*	5	1	4
Bacteraemia	1		1
Central venous catheter	2		2
Local urinary tract infection	1		1
Local infection at site of surgery	1	1	
Fever without neutropenia	1		
Fatigue	12	8	4
Dyspnoea	10	9	1
Nausea	4	3	1
Vomiting	2	1	1
Constipation	1	1	
Diarrhoea	5	4	1
Oral mucositis or candidiasis	4	4	
Rash, maculopapular	7	4	3
Myalgia/arthralgia	1	1	
Petechiae/purpura	1	1	
Atrial fibrillation	1	1	
Delirium	2	2	
Hallucination	2	2	
Hearing impaired	3	2	1
Alopecia	12	12	
Vitiligo	1	1	
Peripheral neuropathy (worsened)	1	1	
Dry skin	2	2	
Rhinitis	1	1	

The table shows treatment-related adverse events according to the CTCAE version 4.0 in all evaluable patients (n = 12).

*Infection verified by microbiological tests.

During hospitalization, expected hematological toxicities attributable to the lymphodepleting chemotherapy regimen were seen (anemia, leukopenia and thrombocytopenia). The number of red blood cell and platelet transfusions, and days with neutrophils<0.5 × 10^9^/L were similar to previously reported data.^6^ All patients suffered from fatigue and/or dyspnoea and nausea at some point either during treatment with chemotherapy and/or IL-2. All patients developed neutropenic fever and were treated with antibiotics. In five patients, an infection was verified by microbial culture; one had bacteraemia and four had local infections at the site of the central venous catheter (n=2), in the urinary tract (n=1), or at the site of surgery (n=1).

IL-2 related toxicity was manageable and intensive care unit intervention was not needed. A median of 96% (range 86–100) of the planned IL-2 dose was administered (online supplemental table S1).

### Clinical efficacy

**Figure 1:**
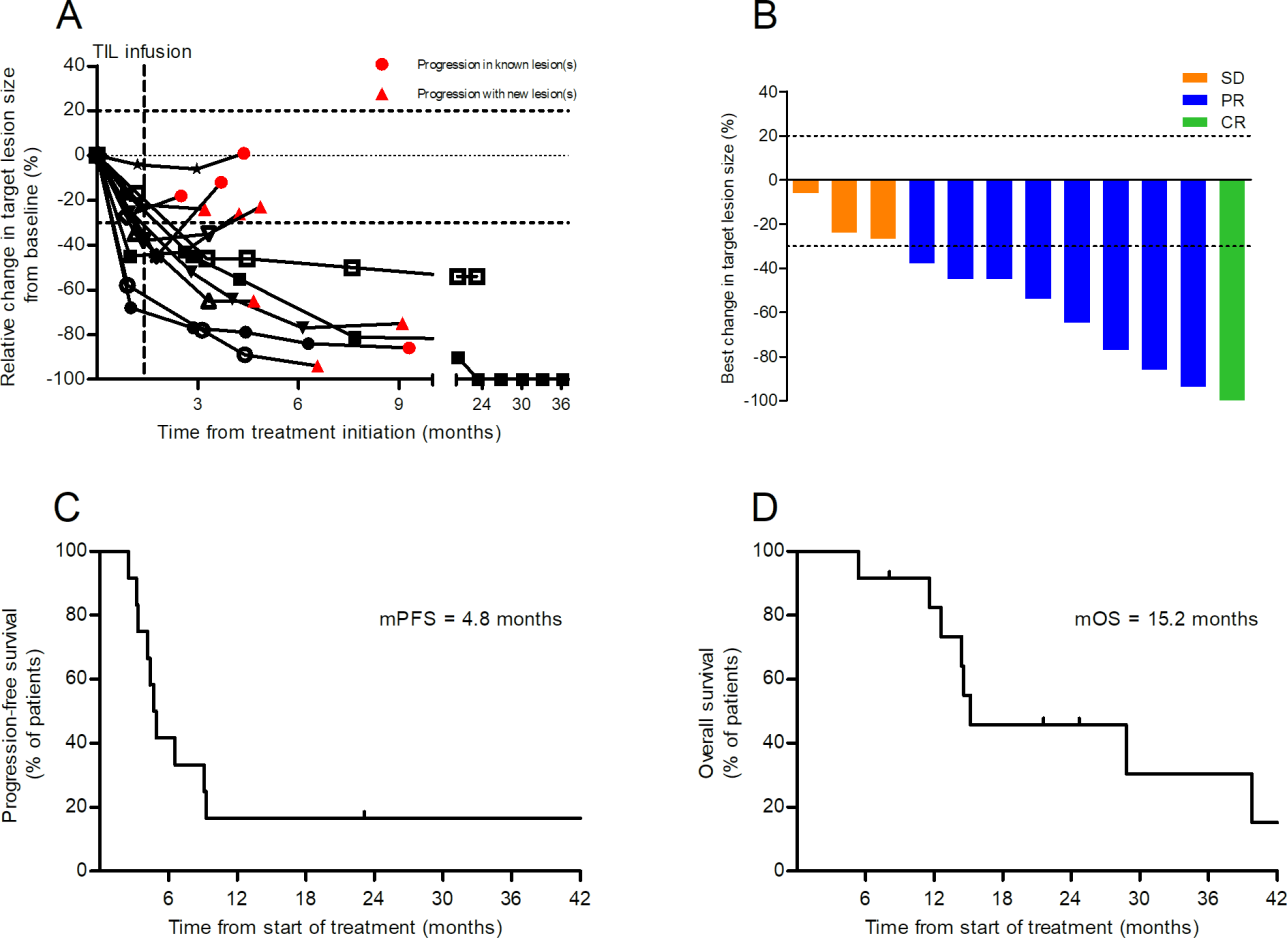
Characteristics of clinical responses and survival. Relative changes in target lesion size from baseline is shown in panel A. Patients marked with red dots had progression of already known tumors whereas patients marked with red triangles had new lesion(s). Vertical line indicates time of infusion of tumor-infiltrating lymphocytes (median 41 days after starting vemurafenib). In panel B, best change in target lesion size during treatment is depicted. Orange bars represents patients with stable disease (SD), blue bars patients with partial responses (PR), and green bar the patient with complete response. In panel C, Kaplan-Meier curves of either progression-free survival (C) or overall survival (D) are shown.

Six of 12 patients obtained a partial response (PR) already during the short-term treatment with vem (patients 02, 05, 06, 09, 10 and 12; figure 1A and table 3). The remaining six had minor tumor regression within the criteria of stable disease (SD). Six patients had further tumor regression fulfilling the criteria for objective response after TIL infusion (patients 03, 04, 05, 06, 07 and 09; marked in bold in table 3), and these patients had longer PFS and OS compared with TIL non-responders (p<0.002 and p=0.006; data not shown). In total, one patient (8.3%; patient 03) achieved a CR, seven patients (58.3%) achieved confirmed PR (one ongoing; patient 07), one had an unconfirmed PR and three patients (25%) had SD as best overall response, as depicted in figure 1B and noted in table 3.

**Table 3:** TIL characteristics and clinical efficacy

Patient	Site of biopsy	TILs cryo before REP	Y-TIL days in culture	Fold Expansion	Infused cells				BOR combined vem +TIL	BOR TIL	PFS (mo)	OS (mo)
					Total (×10^9^)	CD4%	CD8 %	CD8 (×10^9^)				
01	SC	No	34	2200	44	7.3	76.4	33.6	SD	PD	3.2	14.6
02	SC	No	20	4400	88	20.8	69.9	61.5	PR	SD	4.2	12.6
**03**	**SC**	**No**	**21**	**4167**	**125**	**77.3**	**19.5**	**24.4**	**CR (+**)	**CR (+**)	**42.9+**	**42.9+**
**04**	**LN**	**No**	**21**	**2640**	**52.8**	**66.3**	**30.8**	**16.3**	**PR**	**PR**	**9.1**	**39.8**
**05**	**SC**	**Yes**	**13**	**4960**	**99.2**	**18.6**	**75**	**74.4**	**PR**	**PR**	**9.3**	**28.8**
**06**	**SC**	**No**	**29**	**4260**	**85.2**	**12.3**	**87.1**	**74.2**	**PR**	**PR**	**6.6**	**14.4**
**07**	**LN**	**No**	**34**	**2789**	**50.2**	**68**	**19.9**	**10**	**PR (+**)	**PR (+**)	**23.1+**	**24.7+**
**09**	**SC**	**Yes**	**28**	**3250**	**65**	**68.8**	**27.8**	**18.1**	**PR**	**PR**	**4.7**	**21.6+**
10	Lung	No	26	1190	23.8	38	58	13.8	PR	SD	4.9	15.2
11	SC	No	13	4520	90.4	61.3	37.8	34.2	SD	PD	2.5	11.6
12	SC/LN	Yes	25	6160	123.2	5.4	94	115.8	uPR	PD	3.3	5.4
13	SC	Yes	19	5640	112.8	35.5	61.7	69.6	SD	SD	4.4	8.1+
***Median***			***23***	***4213***	***86.6***	***36.8***	***59.9***	***33.9***			***4.8***	***15.2***
***Range***			***(13-34)***	***(2200–6160)***	***(23.8–125)***	***(5.4–77.3)***	***(19.5–94)***	***(10–115.8)***			***(3.2–42.9)***	***(12.6–42.9)***

Plus signs (+) marks ongoing response or survival.

Patients highlighted in bold had further regression after TIL.

BOR, Best overall response according to Response Evaluation Criteria In Solid Tumors version 1.1; CR, complete response; cryo, cryopreserved; LN, lymph node; mo, months; OS, overall survival; PD, progressive disease; PFS, progression-free survival; PR, partial response; REP, rapid expansion protocol; SC, subcutaneous; SD, stable disease; TIL, tumor-infiltrating lymphocytes; uPR, unconfirmed PR; vem, vemurafenib.

The median PFS was 4.8 months, with two patients having ongoing responses for 43 and 23 months (patients 03 and 07, respectively; figure 1C and table 3). In both patients, remaining tumor lesions visible on a CT scan turned FDG-negative after TIL therapy. In three other patients (patients 04, 05 and 09), singular progressive lesions were treated with stereotactic radiotherapy (patient 04) or surgery (patients 05 and 09), and these patients were followed without active systemic treatment for an additional 9, 5 and 6 months until progression in other lesions.

The median OS was 15.2 months (figure 1D). This does also reflect that patients were included before BRAFi treatment and consequently were eligible for treatment with BRAFi alone or in combination with a MEK inhibitor after progression. At the time of data cut-off, five patients were still alive.

### Characteristics of the TIL infusion product

The young TIL cultures were established in a median of 23 days (range 13–34). A median of 86.6×10^9^ TILs (range 23.8–125) were infused on day 0, corresponding to a REP expansion fold of median 4213 (range 2200–6160; table 3). Young TILs were cryopreserved, and subsequently thawed, in four patients before being further propagated in the REP.

**Figure 2:**
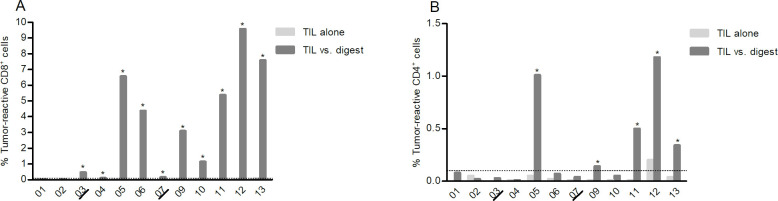
In vitro functionality of infused tumor-infiltrating lymphocyte (TIL). After coculture with autologous tumor digest, tumor reactivity of (A) CD8^+^ and (B) CD4^+^ T cells in the infusion product was assessed measuring interferon-γ, tumor necrosis factor or CD107a by flow cytometry. * indicates in vitro responses, see the Material and methods section for response definition.

The TIL infusion product consisted of a median of 99.1% CD3^+^ cells (range 95.8–99.8). Of those, a median of 36.8% were CD4^+^ and 59.9% were CD8^+^ with high patient variability (table 3). TILs from the infusion product were cocultured with autologous TD. In 10 of 12 patients, a CD8^+^ T cell tumor-specific response was found (figure 2A), and in 5 of 12 a CD4^+^ response was found (figure 2B). The presence of a detectable response in vitro did not correlate with clinical response or PFS (data not shown). However, in vitro response was found in all patients with an objective response to TIL.

### Serum cytokine levels

A multiplexed Luminex assay was performed on serum samples taken at baseline in an exploratory search for cytokine markers predicting response to treatment. Mean baseline levels of IL-6, IL-8, IL-10 and TNF were all numerically lower in patients with a PFS longer than 6 months (after TIL infusion) compared with patients with PFS shorter than 6 months (after TIL infusion; online supplemental figure S3A–D); however, the difference did not reach statistical significance when cytokines were evaluated individually (IL-6: P 0.25; IL-8: P 0.17; IL-10: P 0.09; TNF: P 0.24).

### Tumor biomarker analysis

**Figure 3:**
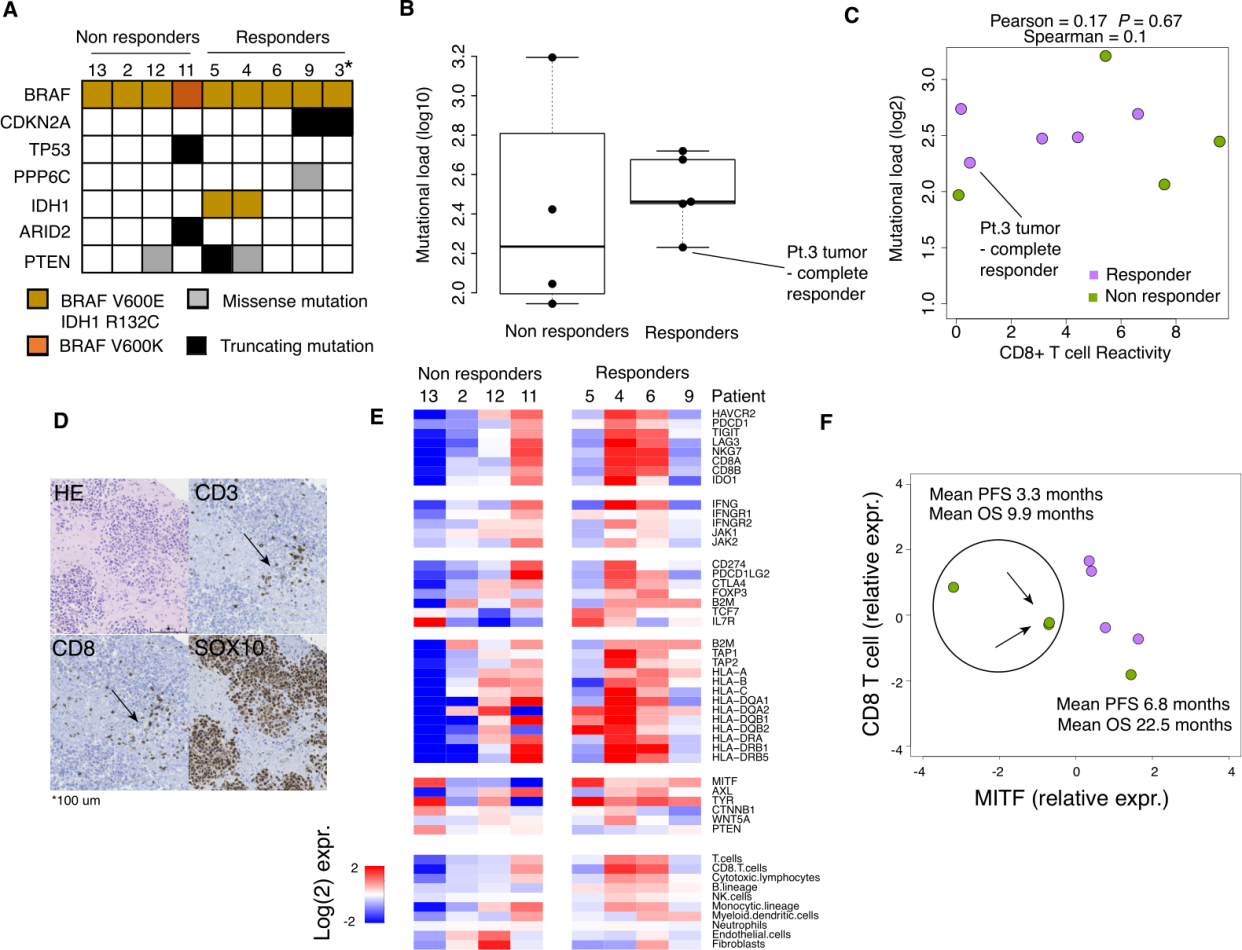
Analysis of tumor genomic properties. (A) Mutation heatmap using data from whole exome sequencing. * indicates patient with complete response. (B) Tumor mutational load between responders and non responders. (C) Tumor mutational load in relation to in vitro measured CD8^+^ T cell reactivity. (D) Representative immunostaining of CD3, CD8, SOX10 and H&E in patient 9. (E) Gene expression heatmap from RNA sequencing data obtained from samples with matched whole exome sequencing data. Selected immune related genes such as T cells specific, interferon gamma signaling, immune checkpoint molecules, antigen presentation and immune evasion are included. Also, the microenvironment cell populations signatures are included. (F) CD8 mRNA in relation to MITF mRNA levels identifies a subset of patients with decreased survival.

A series of biomarker profiles including whole exome-, RNA and TCR sequencing were performed using available patient samples. Nine of the 12 patients had baseline samples obtained after 7 days on vem inhibition with sufficient tumor content to obtain whole-exome sequencing data. Next to the known BRAF mutations, individual samples also harbored mutations in the melanoma driver genes PTEN, CDKN2A and TP53 (figure 3A). Overall, tumors had an average number of 283 (range 88–1564) somatic mutations; however, no difference in tumor mutational burden was found between patients with further tumor regression after TIL infusion (responders) and patients without further regression (non-responders) (p=0.41, figure 3B). Indeed, the tumor sample obtained from the patient achieving CR only harbored 170 somatic mutations. Next, we found no correlation between CD8 T cell tumor-specific response and tumor mutational burden (figure 3C). This suggests that intrinsic tumor genetic alterations are not predictive of response to vem primed TIL therapy. Previous studies have demonstrated that treatment with vem confers an increase in TILs.^18 19^ Our study was not designed to confirm this effect of vem treatment, but the tumors obtained for TIL production was stained with CD3, CD8 and SOX10 antibodies to assess T cell infiltration (figure 3D for representative staining). All tumors had some degree of T cell infiltration, but the infiltration degree was not correlated to therapy response (data not shown).

Next, we obtained gene expression data from tumor specimens from 8 patients using RNA sequencing. Comparing tumors from responder and non-responder samples displayed a high correlation in mean mRNA levels, with only a few genes having a mean log-fold change above two (online supplemental S4A), suggesting that expression levels are not fundamentally different between responders and non-responders. We then extracted genes involved in antigen presentation, interferon-gamma signaling, immune checkpoint molecules, T cell phenotype- and melanoma state markers, and the microenvironment cell population signatures (figure 3E); however, no significant difference was observed. CD8A expression values from tumors were not correlated with levels of tumor-reactive CD8 T cells (online supplemental figure S4B), indicating that the majority of present CD8^+^ cells are part of an unspecific immune response. We then hypothesized that an intrinsic melanoma cell state indicated by MITF mRNA levels and CD8^+^ T cell infiltration in combination is essential for predicting clinical response. When plotting MITF against CD8 mRNA levels we found three tumor specimens clustered on the left side of the plot. These three cases had a mean PFS and OS that were shorter than the samples clustered to the right of the plot. However, the number of cases was too low to reach statistical significance (figure 3F). This is in line with a recent study demonstrating that increase of melanocyte antigens improved response to immune checkpoint blockade.^43^

**Figure 4:**
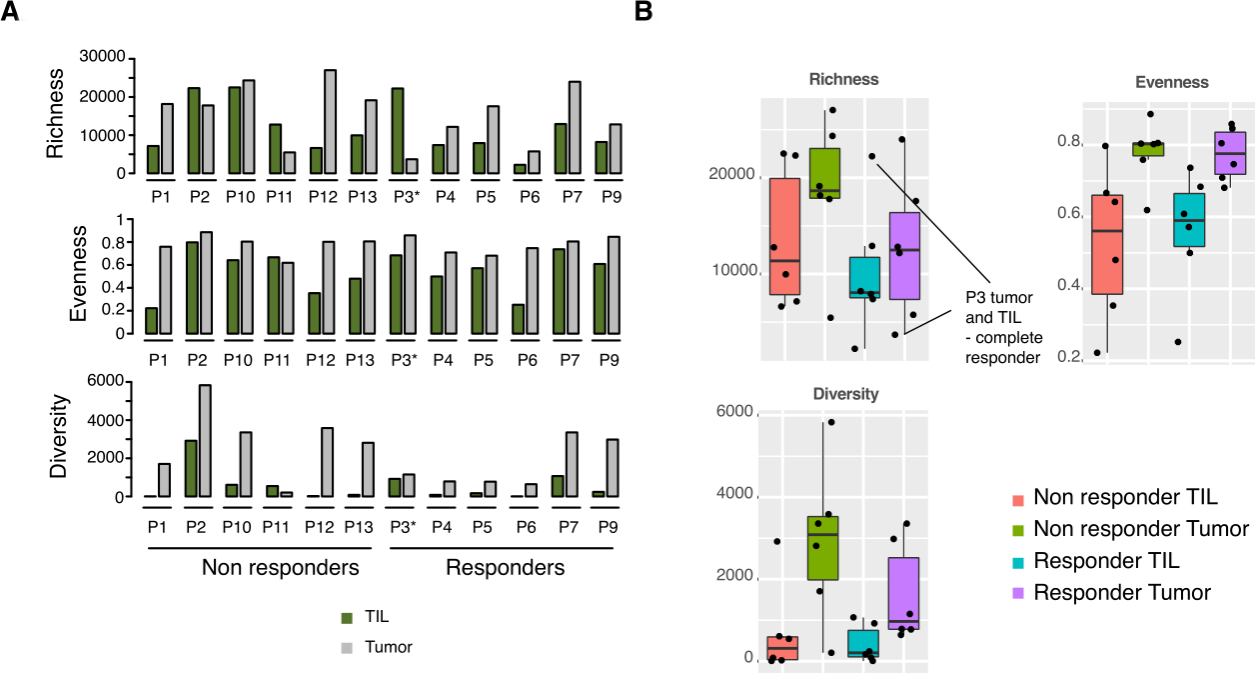
T cell receptor (TCR) sequencing in tumors and tumor-infiltrating lymphocytes (TILs). (A) Individual TCR richness, evenness and diversity values in all patients divided by treatment response. (B) Boxplots of TCR richness, evenness and diversity divided by tumor or TILs and treatment response.

To study the TCR repertoire, we derived TCR sequencing data from tumors removed during vem treatment (n=12) and their corresponding TIL products (n=12). We detected a variable number of clonotypes per sample (ie, richness normalized by the depth of sequencing), defined by different nucleotide TCR sequences: median 12,775.5, range 2223–27 006. We then determined TCR evenness and diversity (figure 4A). There was no correlation between the richness of baseline tumors and TIL products (Pearson 0.05; Spearman 0.03), and there was no significant difference between these two groups (figure 4A). However, intriguingly, evenness was significantly lower in TIL products as compared with tumors (Wilcoxon paired test, p<0.001). We then compared TCR richness, evenness and diversity in TILs and tumor from responders to TILs and tumors from non-responders (figure 4B). Although not significant, the most considerable difference was observed in tumor TCR richness between responders and non-responders with responders having a lower value (figure 4B). Comparison of TCR repertoires of the baseline tumors to their corresponding TIL infusion product revealed low degree of overlap between the repertoires in all patients (online supplemental table S2). In particular, many of the most abundant clonotypes in TIL samples were utterly absent or present at only low frequencies in the corresponding tumors. These data suggest skewing of TCR repertoire during in vitro expansion, promoting certain clonotypes and corroborating a recent study.^44^ In contrast, many of the most abundant T cell clonotypes in tumors were not detected in TIL cultures, suggesting their inefficiency to expand in culture.

### Tumor genomic evolution of melanomas treated with vem and TIL therapy

**Figure 5:**
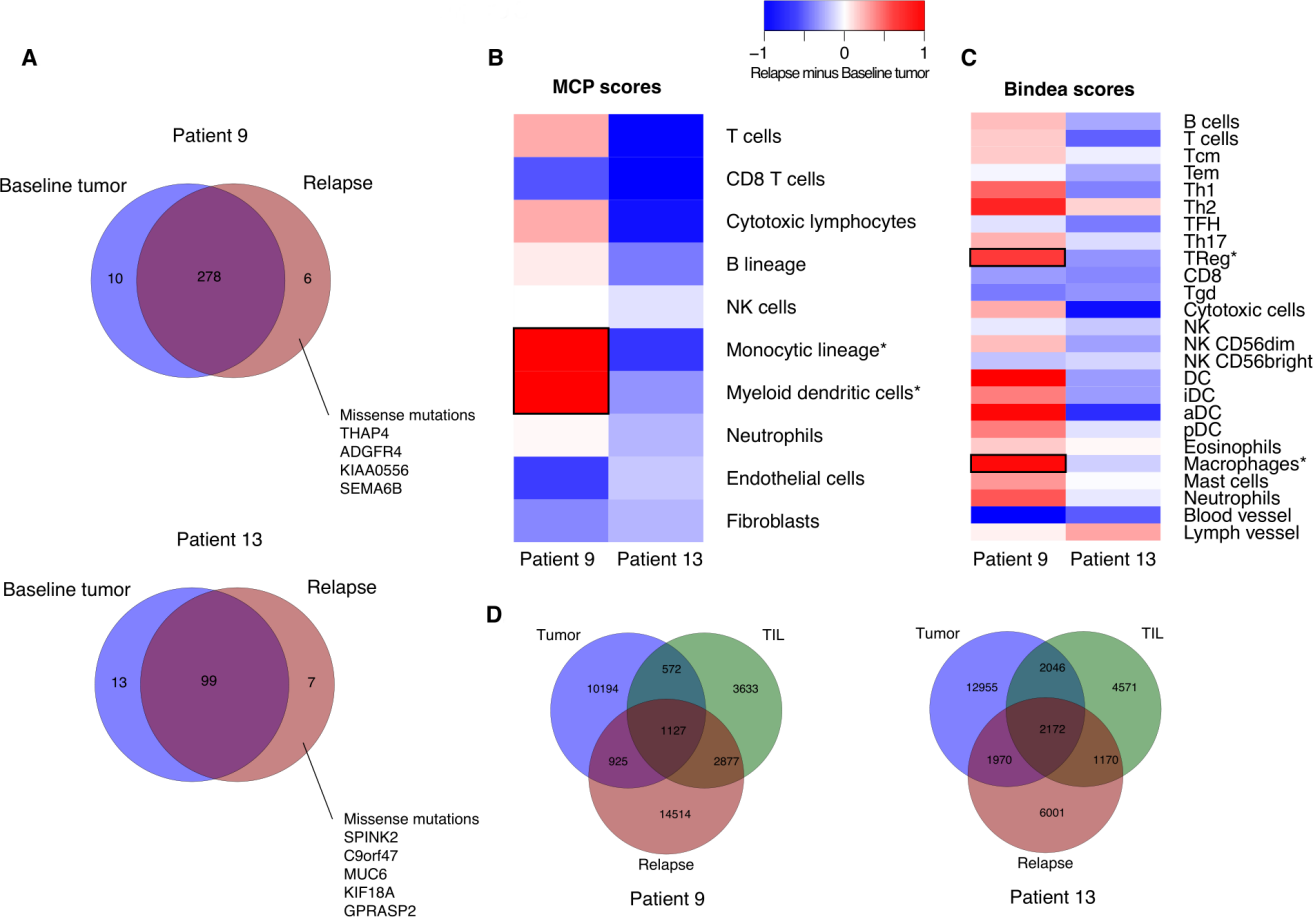
Tumor genomic evolution during vemurafenib and tumor-infiltrating lymphocyte (TIL) therapy. (A) Comparison of whole exome sequencing data from baseline and post relapse tumor samples from two patients. (B, C) Comparison of RNA sequencing derived signatures from baseline and post relapse tumor samples from two patients. Microenvironment cell populations scores (MCP)^47^ and scores using signatures from Bindea *et al*.^46^ Main differences between baseline tumor and post relapse tumor are indicated by a square. (D) Number of T cell clonotypes using T-cell receptor (TCR) sequencing data of baseline and post relapse tumor in comparison to TILs.

To explore the impact of vem primed TIL therapy on tumor evolution, we obtained tumor exome sequencing data during vem treatment and at relapse on TIL therapy from two patients. For both patients, only a limited number of new mutations were detected in the relapse samples (n=4 and n=5 missense mutations for patients 09 and 13, respectively), none of them affecting a known melanoma driver gene (figure 5A). We then compared the transcriptome using predefined signatures for immune cell subsets.^45 46^ In patient 09, the myeloid lineage displayed upregulation in the relapse while in patient 13, a general downregulation of all immune cell subsets in the relapse was found (figure 5B, C). TCR sequencing showed an increase in the number of clonotypes from baseline tumor (12,818) to relapse sample (19,443) with a decrease in evenness (from 0.84 to 0.59) in patient 09, and decrease (from 19 143 to 11 313 clonotypes, with unchanged evenness) in patient 13. The baseline and the relapse tumors from each patient shared few clonotypes, with most of the clonotypes being unique to the particular tumor (figure 5D). Overall, these data show few tumor mutations occurring during resistance development, while TCR repertoire evolves rapidly during evolution.

## Discussion

In this study, we primed the patients with vem before TIL harvest in an attempt to increase quantity and quality of harvested TILs. Patients were kept on vem treatment until TILs were ready for ACT. The aim was to evaluate the safety and feasibility of this approach, as well as the clinical and immunological responses induced by the treatment. We established initial TIL cultures from all patients included, but one patient did not receive TIL infusion due to a progressive CNS metastasis. We did not observe additional toxicity than what has been previously described for either therapies alone,^6 14^ which is in line with findings in two other studies combining vem and ACT using TIL.^24 47^

The manufacturing process of TILs is a long and complicated procedure, with a duration of about 3–6 weeks and with a reported success rate of 65%–95%.^6–9^ Previous studies at our center and elsewhere reported a substantial proportion of patients who clinically deteriorate and/or progress during TIL manufacturing, giving a drop-out rate of more than 30%.^6 7 27^ In this study, the PS of patients was generally stable or improved while on vem treatment during TIL production which is in line with findings reported by Atay *et al*.^24^ Because of the fast induction of tumor regression typically associated with BRAFi, this was largely expected. In this context, BRAFi can function as a bridging therapy allowing TIL therapy for a higher proportion of patients.

In regard to TIL production, we did not observe any benefit from pretreating tumors with vem for 7 days before excision, that is, young TIL culture days, number of TILs infused, CD4/CD8 composition in infusion product, TIL anti-tumor reactivity, compared with what we have previously reported^6^ and another TIL study performed in parallel at our center.^48^ In the study reported by Deniger *et al*,^47^ an additional tumor was excised for immunological studies after 2 weeks of vem treatment in all patients. The group confirmed increased T lymphocyte infiltration into those tumors but did not see an increase in the number of TCR (β subunit) clonotypes nor clonality, and did not find consistent changes in autologous tumor reactivity of TILs grown from vem naïve or pretreated tumors. Of note, anti-tumor reactivity was judged by coculture of TILs with tumor cells obtained from vem naïve tumors, possibly missing the tumor cell-intrinsic added benefit of vem.^22 24 49^

All patients had decreased tumor burden after vem treatment and 6 of 12 had further regression 6 weeks after TIL infusion, indicating an additional effect of TILs in 50% of patients. However, most responses were not long lasting and only two patients have ongoing responses (CR 42.9+months; PR 23.1+months). Thus, these data do not suggest a significantly increased clinical effect of ACT using vem primed TILs, although with this limited cohort, it is impossible to draw firm conclusions. In the clinical trials combining vem and TIL therapy reported by Deniger *et al*^47^ and Atay *et al*,^24^ treatment was sequenced differently, with vem administration starting after surgery and continuing until progression. Although non-randomized trial designs, both trials found no indications of improvement compared with standard TIL therapy regarding ORR and durability of responses.

Before inclusion in our trial, all patients had received treatment with ipilimumab or a PD-1 inhibitor and eight patients had received both as monotherapy. Failure to respond to checkpoint inhibitor therapy suggests a selection of tumors with less favorable histopathological features, i.e. absence of IFNγ signature^50 51^ and dysfunctional antigen processing machinery.^52 53^ We have previously described a high success rate in growing tumor-reactive TILs despite varying presence of T cells in anti-PD-1 resistant tumors.^48^ In a recent publication, we gathered clinical trial data from ten years of TIL trials at our center.^10^ Even in checkpoint-inhibitor resistant MM, we demonstrated a response rate of 32% to TIL therapy, but the duration of response seems to be shorter in patients who previously progressed on PD-1 inhibition. Further, we have recently demonstrated that tumor mutational burden is predictive of response to TIL therapy in anti-CTLA-4 resistant melanoma patients^29^; however, this was not observed in this cohort. Importantly, the current patient cohort includes more clinically challenging patients resistant to multiple immune checkpoint inhibitors. Therefore, there may be a selection of patients with distinct tumor genetic features that are not different between TIL ACT responders and non-responders.

Moreover, immunotherapy response has consistently been associated with a tumor inflammatory phenotype with frequent infiltration of T cells and other lymphocytes.^54 55^ In this study, tumors were excised 7 days after vem treatment started. We found tumor-infiltrating T cells in all tumors on vem treatment although with different degree of infiltration. Neither the degree of infiltration nor the transcriptomic immune signatures were associated with clinical response in our trial. CD8A expression from tumors were also not correlated with autoreactive levels of CD8 T cells suggesting that T cells attracted to the tumor site by vem treatment are not tumor specific. This is further supported by our TCR sequencing analysis in which no significant difference was observed between responders and non-responders. This is contrary to the findings by Riaz *et al*,^56^ which could reflect differences in the cohorts regarding resistance status to checkpoint inhibitors. However, larger clinical cohorts are needed to confirm these results.

The serum analysis performed in this small cohort indicates that baseline levels of cytokines may be useful in predicting long-term outcomes to TIL therapy and warrants further evaluation in larger cohorts. In line with our findings, it was found that IL-2, IL-6, IL-8 and IL-10 were elevated in patients who had a primary melanoma resected, compared with healthy controls.^57^ Further, higher IL-2 and IL-6 levels were correlated to positive sentinel node status. High IL-8 has previously been shown to correlate with tumor burden in animal models and cancer patients,^58^ and a decrease in IL-8 was recently demonstrated to correlate with treatment response to checkpoint inhibitor treatment.^59^ High IL-10 production at the tumor site has been correlated with poor survival,^60^ and increased IL-10 after treatment with a vaccine, correlated with poor survival.^61^

BRAFi treatment is usually coadministered with a MEK inhibitor to improve clinical response rates, delay progression and improve OS.^17^ MEK inhibitors were initially reported to decrease viability and proliferation of (naïve) T cells^13 62^ through abrogation of IL-2 production.^63^ In a later report, this finding was confirmed, but importantly already primed TILs were unaffected.^49^ MEK inhibitor treatment increased CD8^+^ T cell infiltration into tumors, improved T cell function, and partially protected them against activation-induced cell death. It was also shown that melanoma tumor cell lines upregulate melanoma differentiation antigens on stimulation with a MEK inhibitor in vitro regardless of BRAF mutation status.^64^ In light of these observations, adding a MEK inhibitor to the BRAFi treatment or using a MEK inhibitor alone in BRAF wildtype patients might be considered in future TIL-based clinical trials.

In conclusion, we found that priming with vem before TIL harvest and bridging to ACT was safe and feasible. Although clinical efficacy did not seem to improve, vem treatment decreases attrition and could be considered to bridge the waiting time while TILs are prepared.

## Data Availability

Data are available upon reasonable request from the corresponding author.
