# Supplementary material for: Clinical efficacy of T-cell therapy after short-term BRAF-inhibitor priming in patients with checkpoint inhibitor-resistant metastatic melanoma
Source: J Immunother Cancer. 2021 Jul 1;9(7):e002703. doi: 10.1136/jitc-2021-002703 (PMC8252872; doi:10.1136/jitc-2021-002703)
Supplement: Supplementary data [file jitc-2021-002703supp001.pdf]

Supplementary material for

Clinical efficacy of T-cell therapy after short-term BRAF-inhibitor priming in patients with checkpoint inhibitor resistant metastatic melanoma

Borch TH, *et al. J Immunother Cancer* 2021; 9:e002703. doi: 10.1136/jitc-2021-002703

Supplementary table S1. Treatment characteristics						
**Patient**	**Days on vemurafenib**	**Days in hospital[a]**	**Units RBC transfusion**	**Units PLT transfusion**	**Neutrophils < 0.5 x 10^9/l (days)**	**IL-2 dose MIU (%[b])**
01	47	17	0	7	4	85.1
02	33	16	3	7	7	95.2
03	34	20	4	11	9	100
04	34	17	1	2	7	100
05	35	19	1	7	8	85
06	42	19	3	6	10	100
07	47	17	3	4	7	100
09	50	20	8	7	13	90
10	49	23	5	9	10	98
11	26	18	9	8	8	97
12	61	18	3	6	7	90
13	40	15	3	1	5	97
Median	***41***	***18***	***3***	***7***	***7***	***96***
Range	***(26-61)***	***(15-23)***	***(0-9)***	***(1-11)***	***(4-13)***	***(85-100)***

Abbreviations: MIU, million International Units; RBC, red blood cell; PLT, platelet. [a]Length of hospitalization due to T-cell therapy. [b]Percentage administered of intended dose.

Supplementary table S2. TCR repertoire overlap of tumors (TUM) and TIL products				
**Patient**	**TUM total number of clonotypes[1]**	**TIL total number of clonotypes[1]**	**Shared clonotypes**	**Morisita-Horn index**
01	18160	7154	2030	0.017
02	17787	22317	2738	0.316
03	3690	22233	1652	0.171
04	12173	7398	2497	0.059
05	17571	7920	3672	0.227
06	5750	2231	734	0.098
07	23994	12915	4451	0.236
09	12818	8209	1699	0.018
10	24347	22510	5354	0.025
11	5478	12776	2222	0.611
12	27022	6641	2845	0.006
13	19143	9959	4218	0.016

[1] Actual detected clonotype numbers, non-normalized for between-samples comparison

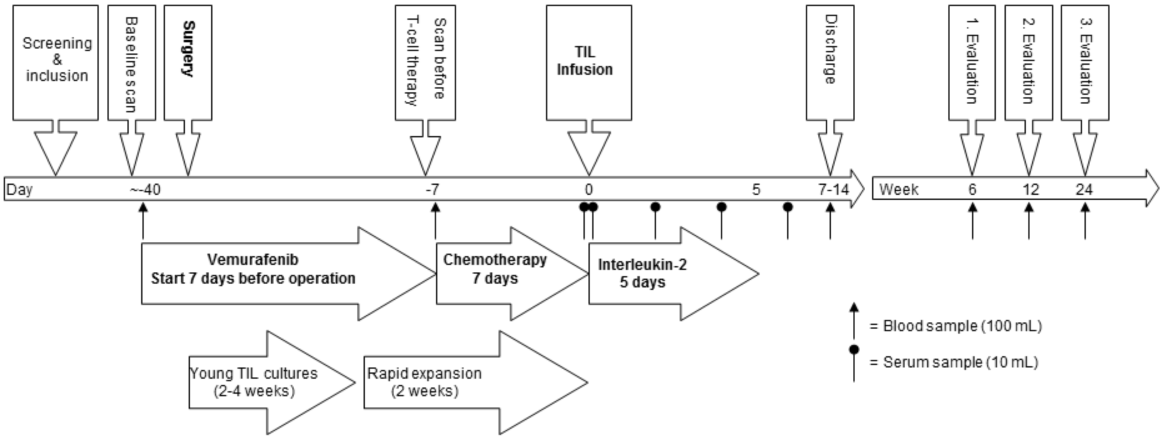

Supplementary figure S1. Treatment and monitoring schedule

After screening and inclusion the patient received vemurafenib treatment for seven days prior to tumor excision and continuously until admission for T-cell therapy. Excised tumor lesions were cut into fragments and placed in culture plates with interleukin-2 (IL-2) containing medium. Tumor-infiltrating lymphocytes (TIL) were expanded following the young TIL method until a minimum of 50 x 10^6 cells are obtained, usually within 2-4 weeks. TILs were further propagated in the 14 days rapid expansion protocol.

Meanwhile the patient was hospitalized in order to receive a preconditioning lymphodepleting chemotherapy regimen consisting of two days of cyclophosphamide and five days of fludarabine. After TIL infusion (day 0), a continuous IL-2 infusion following the decrescendo-regimen was administered. The patient was discharged after hematological and clinical recovery (7-14 days after TIL infusion).

PET/CT-scans were performed before treatment initiation, before admission and at subsequent evaluations. Blood and serum samples for immune monitoring were collected before, during and after treatment as indicated.

Borch TH, *et al. J Immunother Cancer* 2021; 9:e002703. doi: 10.1136/jitc-2021-002703

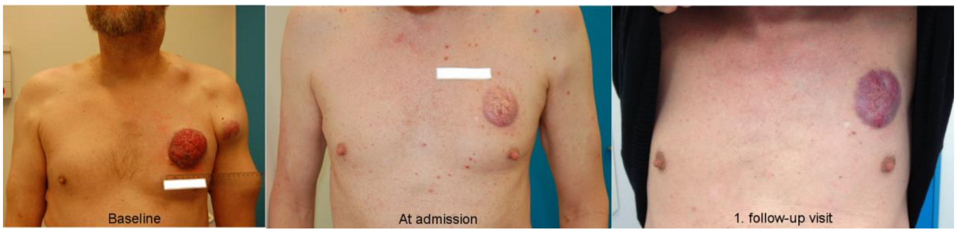

Supplementary figure S2. Clinical photos

Clinical photos of a patient showing bulky cutaneous and subcutaneous tumors. The rapid effect of vemurafenib is evident as tumors are visibly smaller after 33 days treatment. At the first follow-up visit tumors are roughly the same size, but appear more pigmented. Progression is confirmed at the following visit with radiological evidence of new lesions.

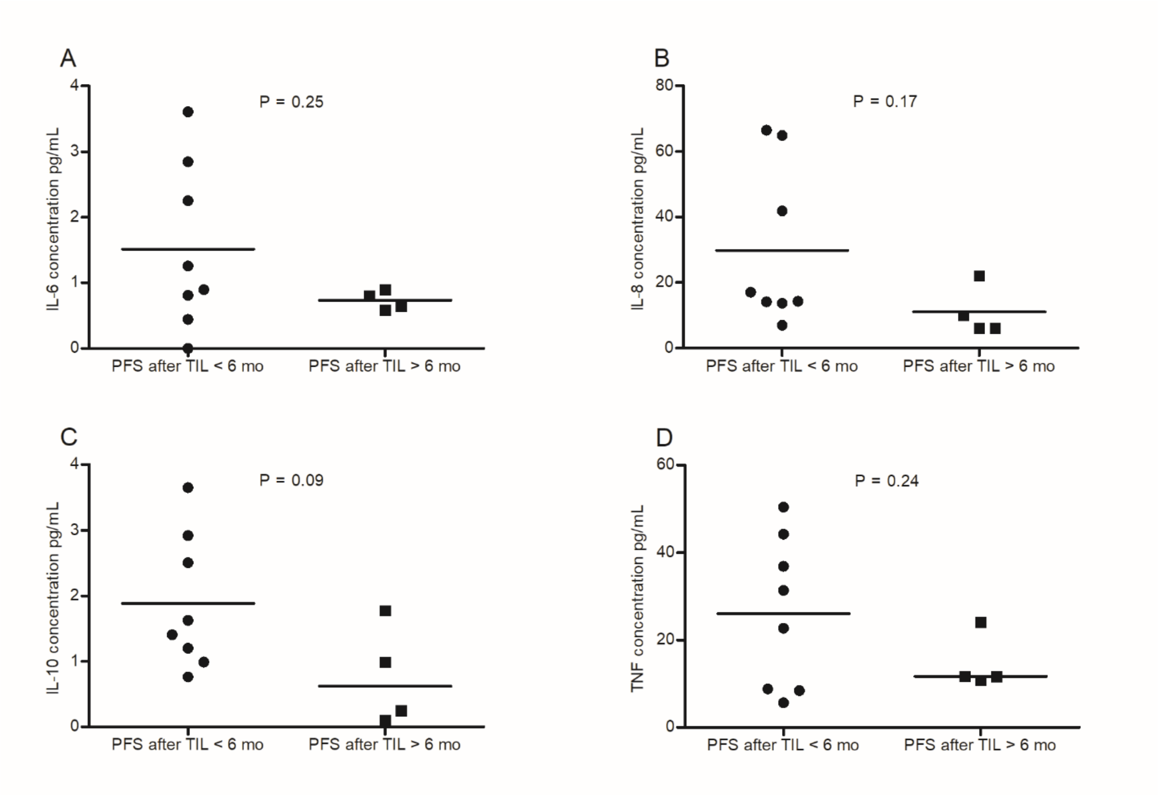

Supplementary figure S3. Baseline cytokines levels

The figures depict serum concentrations of interleukin-6 (IL-6; A), IL-8 (B), IL-10 (C) and tumor necrosis factor (TNF; D) for individual patients (n = 12). All of the cytokines show a trend towards higher levels correlating with shorter progression-free survival (PFS). Each symbol represents mean value of doublet measurements in each patient. Horizontal lines represent mean values. (Mo) denotes months.

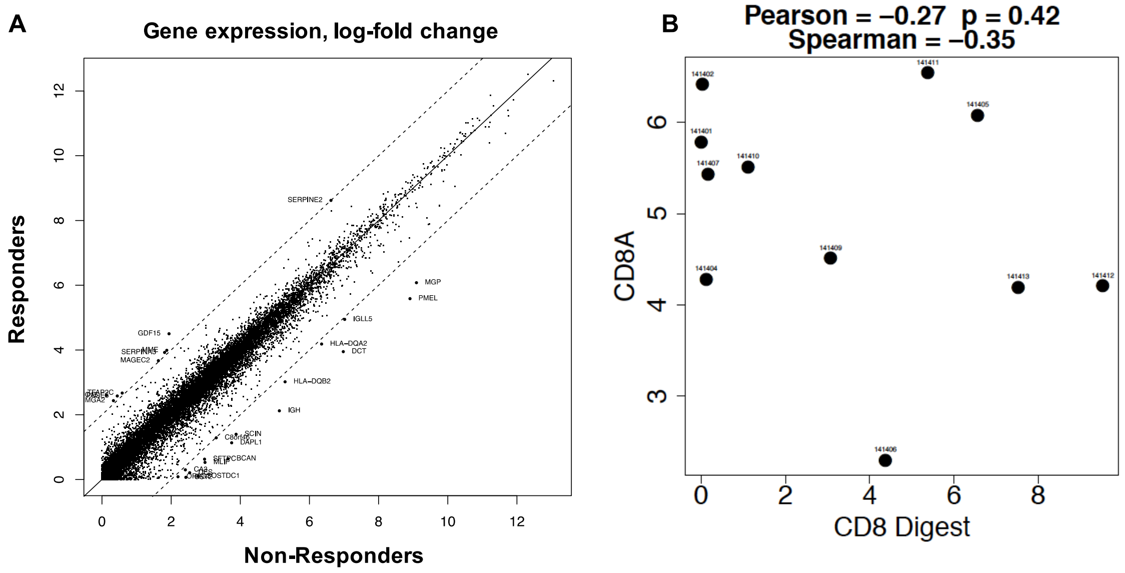

Supplementary figure S4.

A. Mean gene expression of Responders and Non-Responders. Dotted line represents a log-fold change of 2. **B**. Correlation of CD8A gene expression to the in vitro reactivity of the CD8 T cells to the autologous tumor digest.

Borch TH, *et al. J Immunother Cancer* 2021; 9:e002703. doi: 10.1136/jitc-2021-002703
